# Estimating the Complete Basis Set Extrapolation Error through Random Walks

**DOI:** 10.1021/acs.jpclett.5c00749

**Published:** 2025-05-12

**Authors:** Jakub Lang, Michał Przybytek, Michał Lesiuk

**Affiliations:** University of Warsaw, Faculty of Chemistry, Pasteura 1, 02-093 Warsaw, Poland

## Abstract

We propose a method for estimating the uncertainty of a result obtained through extrapolation to the complete basis set limit. The method is based on an ensemble of random walks that simulate all possible extrapolation outcomes that could have been obtained if results from larger basis sets had been available. The results assembled from a large collection of random walks can then be analyzed statistically, providing a route for uncertainty prediction at the confidence level required in a particular application. The method is free of empirical parameters and compatible with any extrapolation scheme. The proposed technique is tested in a series of numerical trials by comparing the determined confidence intervals with reliable reference data. We demonstrate that the predicted error bounds are reliable and tight yet conservative at the same time.

## Graphical abstract

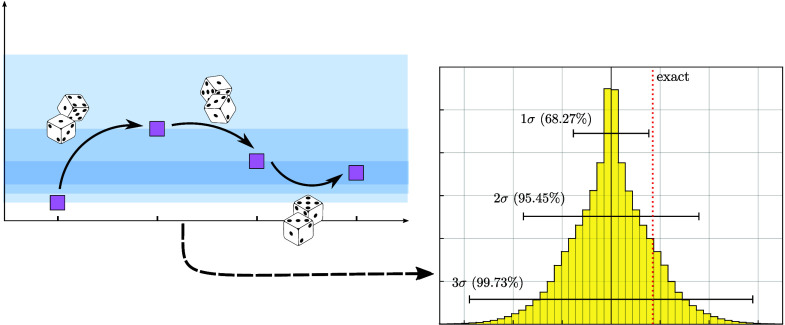

The demand for accurate quantum-chemical calculations for many-electron atoms and molecules has been rapidly increasing in recent years, fueled by developments in fields such as ultracold chemistry and physics,^1–5^ quantum-based metrology,^6–11^ spectroscopy,^12–16^ or search for effects beyond the standard model.^17–21^ It is striking that in a vast majority of these studies, it is necessary to provide not only accurate theoretical results that account for all relevant physical effects but also to estimate the uncertainty of the calculated data. Simultaneously, most calculations of this type employ a basis set for expansion of spin-orbitals/spinors, which naturally leads to an error that must be controlled. It is well-known that due to the electronic cusp condition,^22,23^ the results of correlated calculations converge slowly with respect to the basis set size. Consequently, the development of methods that reduce the basis set incompleteness error remains an active field of research. Explicitly correlated methods,^24–26^ transcorrelated approaches,^27^ density-based corrections,^28–31^ and extrapolation techniques^32–35^ are frequently applied for this purpose. In this paper, we focus on the last family of methods.

Extrapolation to the complete basis set limit is an attractive option of reducing the basis set incompleteness error due to its conceptual simplicity, vanishingly small computational cost, and broad applicability. Several extrapolation methods are frequently used in the literature, and there is general consensus that, when used with care, they considerably improve the results (see, for example, ref 34 for a detailed analysis). However, the estimation of the uncertainty of the extrapolated results and determination of proper error bars are challenging issues with no general guidelines available. Assignment of the uncertainty is usually based on, for example, comparing extrapolated results from a progression of basis sets,^10,14^ applying different extrapolation schemes and observing variation between them,^21,36^ or comparing the extrapolated result with the value obtained with the largest available basis set.^2,37^ Alternatively, comparison with external reference data, either theoretical or experimental, is an option for selecting the proper extrapolation protocol, but such data may not be available in many situations. In any case, the estimation of the residual extrapolation error frequently involves a degree of arbitrariness or secondary assumptions.

Another problem related to this issue, which is particularly important at the interface of theory and experiment, is the different meaning of the uncertainties in these fields. In the experiment, one typically repeats the same measurement numerous times and assumes that the variation in the data is represented by a certain probability distribution. The uncertainties are then assigned based on confidence intervals resulting from this distribution, leading to a clear statistical meaning of the error bars. Such a procedure is usually impossible in theory, and hence, the meaning of the error bars assigned to a theoretical result is simply a statement that with a sufficiently high probability, the exact result differs from the calculation by less than a certain value. However, it is typically not known what this probability really is, and there is no way of tracing it back to any confidence interval based on statistical analysis. Of course, it is also possible to compare various extrapolation schemes by benchmarking against a set of reference data,^34^ but there is no guarantee that the conclusions can be transferred to a particular problem at hand that is outside the training set. In other words, this approach is inherently not system-specific.

In this work, we propose a method of assigning uncertainties to theoretical results obtained by extrapolation. The method is based on a series of random walks that simulate possible results that could have been obtained if data calculated with larger basis sets had been available. While a single random walk does not carry any practical information, an ensemble of random walks can be analyzed statistically to uncover the possible variation in the extrapolated results. This provides a route for uncertainty prediction without arbitrary assumptions in a system-specific way.

In order to introduce the proposed method, let us consider calculation of a certain quantity *E* using a progression of basis sets^38^ and subsequent extrapolation of the results to the complete basis set limit. The size of the basis set is denoted by a single parameter *X* (for example, cardinality in the case of correlation-consistent basis sets^39^). The value of *E* calculated within basis set *X* is denoted by the symbol *E*_*X*_. For a sufficiently large *X*, we can expand *E*_*X*_ in the asymptotic series:

1 $$E_{X}=E_{\infty }+\overset{\infty }{\underset{n=3}{\sum }}\frac{A_{n}}{X^{n}}$$

It is well-known that in the case of electronic energy and many other quantities, the dominant term of this expansion is proportional to *X* ^–3^ (see refs 40–43 ). Many extrapolation procedures use this information either directly or implicitly. In this work, we shall employ primarily the two-point extrapolation scheme of Helgaker et al.,^44,45^ which is based on truncating the above expression after the leading-order term, i.e., $E_{X}=E_{\infty }+\frac{A_{3}}{X^{3}}$ . Next, the results obtained with two consecutive basis sets, *E*_*X*_ and *E*_*X*–1_, are combined to eliminate the *A*_3_ coefficient. This gives the following explicit formula for the estimate of the complete basis set limit:

2 $$E_{\infty }\approx \frac{E_{X}X^{3}-E_{X-1}{\left(X-1\right)}^{3}}{X^{3}-{\left(X-1\right)}^{3}}$$

Let us denote the value extrapolated according to eq 2 from the pair of basis sets (*X*, *X* – 1) by the symbol *e*_*X*_.

Estimation of the extrapolation error is a difficult task primarily because (i) little is known analytically about the higher-order terms in eq 1 and coefficients *A*_*n*_ for many-electron systems, (ii) the accessible range of *X* is typically too narrow to determine them reliably, e.g., by fitting, and (iii) secondary sources of error such as radial incompleteness may play a role for any finite *X*. In this work, we adopt a minimalist assumption about the behavior of *e*_*X*_ as a function of *X*. We assume only that the absolute differences between neighboring extrapolated values, |*e*_*X*_ – *e*_*X*–1_|, decrease monotonically for a sufficiently large *X*, but *e*_*X*_ themselves do not need to follow any consistent pattern. For example, in the case of the extrapolation of formula [Disp-formula eq2], one can show that these differences behave for large values of *X* as

3 $$e_{X}-e_{X-1}=\frac{C}{X^{5}}+...$$

where C is a system-dependent numerical constant and the higher-order terms (proportional to *X* ^–*n*^ with *n* ≥ 6) are not written explicitly. From this formula, it is evident that the quantities |*e*_*X*_ – *e*_*X*–1_| decrease monotonically for a sufficiently large *X*, even if *e*_*X*_ themselves do not exhibit a monotonic behavior, e.g., oscillate. Note that the value of C could, in principle, be obtained by using results from a progression of basis sets, but we found that such an approach is not trustworthy when applied within the range of *X* that is typically available.

Let us assume that we carried out calculations within three consecutive basis sets (*X*, *X* – 1, and *X* – 2), while results for larger basis sets are not available. From these data, we can assemble two extrapolated values, *e*_*X*_ and *e*_*X*–1_. According to our main assumption, if the next extrapolated value (*e*_*X*+1_) had been available, it would have been bounded by

4 $$e_{X}-\left|e_{X}-e_{X-1}\right|<e_{X+1}<e_{X}+\left|e_{X}-e_{X-1}\right|$$

At face value, this inequality in itself is not very useful because we do not know the actual value of *e*_*X*+1_. More importantly, there is no guarantee that the exact result (*E*_∞_) also lies within this interval. However, we can pessimistically assume that any value of *e*_*X*+1_ within the bounding interval is equally probable and randomize it from a uniform distribution. In this way, we obtain a value of *ẽ*_*X*+1_ that represents one possible scenario of what the actual *e*_*X*+1_ may be. This procedure is then continued. Assuming the randomized value of *ẽ*_*X*+1_, we know that the next extrapolated value (*e*_*X*+2_) is bounded by

5 $${ẽ}_{X+1}-\left|{ẽ}_{X+1}-e_{X}\right|<e_{X+2}<{ẽ}_{X+1}+\left|{ẽ}_{X+1}-e_{X}\right|$$

and again randomize *ẽ*_*X*+2_ within this interval. This procedure eventually converges in the sense that after a certain number of steps, *N*, the length of the bounding interval becomes smaller than a predefined threshold. At the same time, two successive randomized values (*ẽ*_*X*+*N*_ and *ẽ*_*X*+*N*–1_) obviously differ by less than this threshold. In the following, we refer to the set of *ẽ*_*X*+1_, *ẽ*_*X*+2_, ..., as a trajectory and denote converged value *ẽ*_*X*+*N*_ by *ẽ*_∞_.

A single trajectory in the proposed method is essentially a random walk, where the values of *ẽ*_*X*+1_, *ẽ*_*X*+2_, ..., are allowed to randomly shift within the corresponding bounding intervals. However, we stress that a single trajectory obtained in this way is not useful for any practical purpose. It represents only one possible scenario of what could have happened if results in larger basis sets had been available (having access to the subsequent extrapolated results *e*_*X*+1_, *e*_*X*+2_, ...). The proposed method becomes useful only when a large number of trajectories is run independently. It provides insight into the variability of *ẽ*_∞_ without any assumptions about the particular values of *e*_*X*+1_, *e*_*X*+2_, ... The only assumption used in this procedure is the monotonic decrease in the absolute differences between extrapolated values as a function of *X*. Having a large number of *ẽ*_∞_ obtained from separate trajectories, the results can be analyzed statistically. This naturally leads to system-specific uncertainty estimates for the average value of *ẽ*_∞_, as demonstrated below.

Let us first illustrate the proposed method by applying it to two model systems for which both reliable reference data and results obtained within a progression of basis sets are available. Our main goal here is a detailed discussion of the algorithm of the proposed method, while the presentation of results for a much larger set of systems is given later. The first example is the electronic correlation energy of the H_2_ molecule (internuclear distance of 1.4 au) calculated within aug-mcc-pVXZ basis sets of Mielke et al.^46^ using the full configuration interaction (FCI) method. The reference values for the total and Hartree–Fock energies of H_2_ come from the work of Pachucki^47^ and Mitin,^48^ respectively, giving the near-exact value of the correlation energy equal to −40.846 348 mHa. The second example is the correlation energy of the carbon atom calculated at the FCI/aug-cc-pCVXZ^49^ level of theory. Based on accurate results for the total energy obtained by Strasburger^50^ and the Hartree–Fock energy by Bunge et al.,^51^ the reference value for the correlation energy is −156.287 mHa. In the first example, results within basis sets up to *X* = 6 are available, while for the second example, we are limited to *X* = 4. The test cases were purposefully chosen to study the performance of the proposed method in these two distinct situations, both of which are frequently encountered in practice. The raw results used in our analysis were calculated in ref 52 and are reproduced in Table 1 for the sake of convenience.

**Table 1:** Raw Data and a Summary of the Results for Two Selected Test Cases^a^

	test case 1 (H_2_ molecule)		test case 2 (carbon atom)	
*X*	–*E*_*X*_	–*e*_*X*_	–*E*_*X*_	–*e*_*X*_
2	–	–	132.539	–
3	–	–	145.934	151.574
4	40.6528	–	151.029	154.747
5	40.7374	40.8262	–	–
6	40.7797	40.8378	–	–
Best Estimates				
1σ (68.27%)	40.8378 ± 0.0078		154.7 ± 2.2	
2σ (95.45%)	40.838 ± 0.018		154.7 ± 4.8	
3σ (99.73%)	40.838 ± 0.029		154.7 ± 7.9	
true error^a^	0.0085		1.540	
reference	40.8463		156.287	

a See the text for computational details. All values are given in mHa (with signs reversed for the sake of convenience).

b Absolute deviation from the reference data given in the last row.

The random walk procedure was initiated using the extrapolated value from the pair of the two largest basis sets. However, results from three consecutive basis sets are necessary to establish the initial bounding interval (see eq 4). About 10 million trajectories were simulated in both test examples; a further increase in this parameter leads to no appreciable changes in the uncertainty predictions. The values of *ẽ*_*X*_ were randomized from a uniform distribution. Each random walk was stopped when the width of the bounding interval (see eqs 4 and 5) falls below the threshold of 10^–16^. The converged values of *ẽ*_∞_ for each trajectory were recorded and are the subject of the analysis that follows.

In Figure 1, we provide histograms illustrating the distribution of *ẽ*_∞_ obtained after about 10^7^ random walks. The distributions are nearly symmetric with respect to the mean, which is not surprising considering that the end points of the bounding intervals (see eqs 4 and 5) are always equidistant from the previous extrapolated value. For the same reason, as the sample size increases the average value of *ẽ*_∞_ obtained from all walks should converge to extrapolated result *e*_*X*_ that was used to initiate the random walks (see eq 4 and the accompanying discussion). This is confirmed in our calculations, with agreement of six significant digits in all cases. Therefore, we reiterate that the method proposed in this work enables us to estimate the uncertainty of an extrapolated result, while the result itself is unchanged in comparison with the value of *e*_*X*_ used to initiate the random walks (see Table 1).

**Figure 1:**
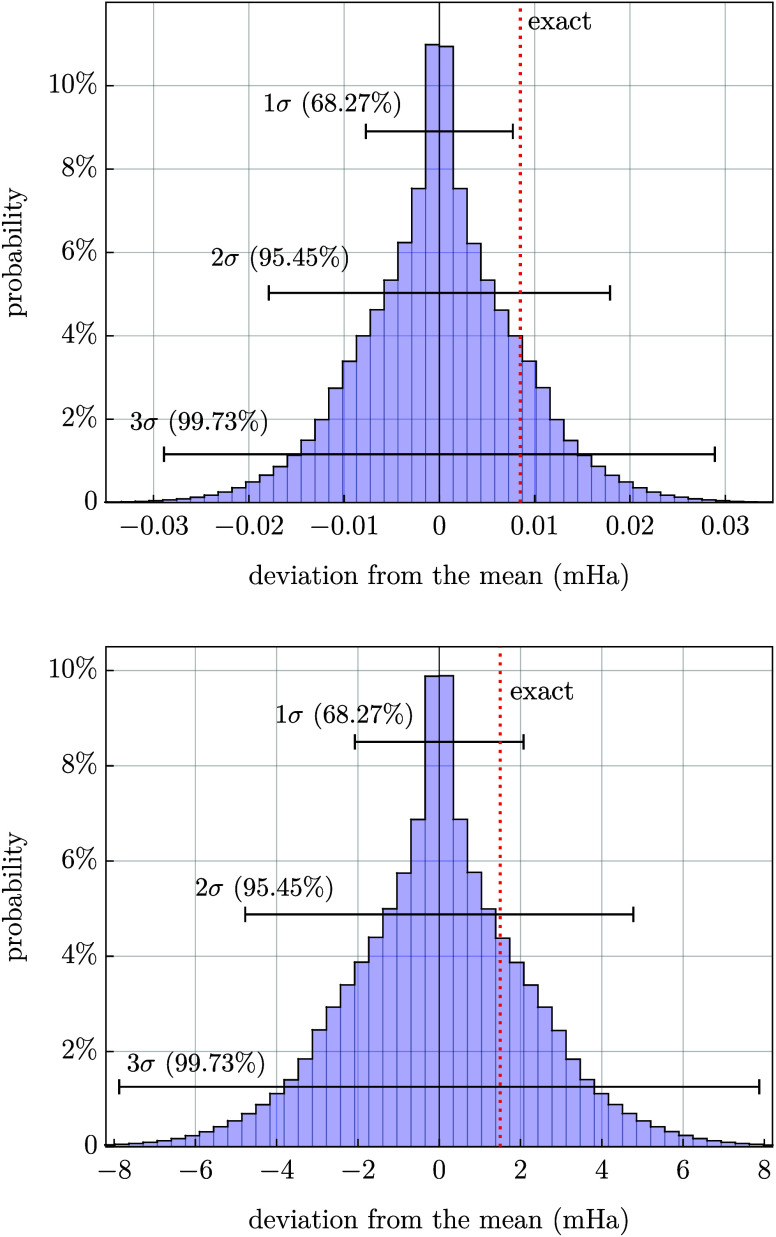
Histograms illustrating the results of about 10^7^ random walks for test case 1 (top) and test case 2 (bottom). The histograms are centered such that the sample mean corresponds to zero at the horizontal axis. The deviations from the mean are given in mHa. The 1σ, 2σ, and 3σ confidence intervals (see the text) are shown as overlaying brackets. The reference (near-exact) values are represented as red dotted lines.

The probability distributions represented in Figure 1 enable us to assign confidence intervals to the extrapolated results. We consider three confidence intervals at confidence levels of 68.27%, 95.45%, and 99.73%. The choice of these percentages is arbitrary and is motivated by analogy to the commonly used values in the case of a normal distribution. However, we stress that the probability distributions obtained in the present context are clearly not normal, and hence, the lengths of the confidence intervals are not simple multiples of the standard deviation calculated from the sample, as in the case of the Gaussian distribution. Instead, the confidence intervals are defined as intervals centered at the sample mean that cover a given percentage of the data points, as illustrated in Figure 1. For brevity and by analogy with the normal distribution, we refer to the confidence intervals at confidence levels of 68.27%, 95.45%, and 99.73% as 1σ, 2σ, and 3σ, respectively. The confidence intervals determined by the proposed procedure for the H_2_ molecule (test case 1) and carbon atom (test case 2) are shown in Table 1. In both cases, they successfully estimate the extrapolation error. In the former case, the 2σ confidence interval correctly predicts the deviation from the reference value, while in the latter, even the 1σ confidence interval is sufficient for this purpose.

As a side note, we mention that according to the numerical tests, the probability distributions shown in Figure 1 do not seem to be well represented by a simple analytic form such as the Laplace (bivariate exponential) distribution. We were not able to find the exact analytic form of this distribution in the limit of the infinite number of independent trajectories. Mathematically, this is a difficult task, because the randomization steps involved in a single trajectory are strongly interdependent; i.e., the interval in which the subsequent randomization is performed depends directly on the result of two previous samplings. From a pragmatic standpoint, the lack of this information is not problematic, because the computational cost of running a single trajectory is very low. Therefore, assembling a sufficient number of samples for a credible statistical analysis is not challenging. Calculations with 10 million random walks take mere seconds.

To illustrate the performance of the proposed method for a larger set of examples, we gathered numerous results from the literature in which results of the calculations from a progression of basis sets are available and, simultaneously, reliable reference data are found. The main sources of the reference values are either explicitly correlated calculations (explicitly correlated Gaussians^53,54^ or the F12 methodology^24–26^ ) or calculations with basis sets significantly larger than those used in the error estimation procedure. The benchmark set includes both correlation energies, given in Table 2, and other quantities, such as atomization energies, interaction energies, or polarizabilities, given in Table 3. In all cases, around 10 million trajectories were run, which is sufficient to make the confidence intervals stable to all digits shown (as a rule, the last digit has always been rounded up).

**Table 2:** Estimated Extrapolation Errors for Correlation Energies of a Benchmark Set of Systems^a^

				confidence intervals				
system	method and basis	*X*_max_	*e*_*X*_max__	1σ (68.27%)	2σ (95.45%)	3σ (99.73%)	error	reference value
He atom	FCI	4	–41.907	±**0.17**	±0.37	±0.62	0.137	–42.044 381^a^ ^,^ ^56,57^
	d*X*Z^55^	5	–41.983	±0.050	±**0.12**	±0.19	0.062	
		6	–42.013	±0.020	±**0.045**	±0.074	0.032	
		7	–42.026	±0.009	±**0.021**	±0.034	0.018	
Be atom	FCI	4	–94.099	±0.20	±**0.46**	±0.76	0.233	–94.332 459^b^ ^,^ ^58^
	Slater-type basis^12^	5	–94.253	±**0.11**	±0.23	±0.39	0.079	
		6	–94.305	±**0.035**	±0.078	±0.13	0.027	
Be atom	MP2	4	–75.711	±**1.2**	±2.7	±4.4	0.648	–76.358^c^ ^,^ ^59^
	aug-cc-pwCV*X*Z^52^	5	–76.085	±0.25	±**0.56**	±0.93	0.274	
Be atom	CCSD	4	–93.633	±**0.59**	±1.4	±2.2	0.031	–93.665^d^ ^,^ ^59^
	aug-cc-pwCV*X*Z^52^	5	–93.586	±0.032	±0.071	±**0.12**	0.079	
H_3_^+^ cation	FCI	4	–43.432	±**0.062**	±0.14	±0.24	0.032	–43.464^e^ ^,^ ^60,61^
	aug-mcc-pV*X*Z^52^	5	–43.441	±0.007	±0.014	±**0.024**	0.023	
LiH molecule	MP2	4	–72.343	±**1.5**	±3.2	±5.3	0.546	–72.890^f^ ^,^ ^62^
	aug-cc-pwCV*X*Z^52^	5	–72.660	±0.21	±**0.48**	±0.79	0.230	
LiH molecule	CCSD	4	–83.103	±**0.27**	±0.60	±1.0	0.113	–82.990^f^ ^,^ ^62^
	aug-cc-pwCV*X*Z^52^	5	–82.623	±0.32	±**0.72**	±1.2	0.367	
Ne atom	frozen core (1s^2^)	4	–315.628	±**13**	±28	±46	4.595	–320.223^g^ ^,^ ^65,66^
	MP2/*X*ZaP^63,64^	5	–319.003	±**2.3**	±5.1	±8.4	1.220	
		6	–319.600	±0.40	±**0.90**	±1.5	0.622	
		7	–319.881	±0.19	±**0.42**	±0.70	0.342	
		8	–319.985	±0.070	±0.16	±**0.26**	0.238	
		9	–320.073	±0.059	±0.14	±**0.22**	0.150	
Ne atom	(T) correction	4	–6.535	±**0.60**	±1.4	±2.3	0.038	–6.497^h^ ^,^ ^64^
	*X*ZaP basis^64^	5	–6.647	±0.075	±**0.17**	±0.28	0.150	
		6	–6.554	±**0.062**	±0.14	±0.24	0.057	
		7	–6.530	±0.016	±**0.035**	±0.058	0.033	
		8	–6.518	±0.008	±0.018	±**0.030**	0.021	
H_2_O molecule	MP2	4	–298.39	±**7.8**	±18	±29	1.96	–300.35^i^ ^,^ ^32^
	cc-pV*X*Z^32^	5	–300.67	±**1.6**	±3.4	±5.7	0.32	
		6	–300.29	±**0.26**	±0.57	±0.95	0.06	
CH_2_ molecule	MP2	4	–155.08	±**3.0**	±6.7	±11	0.73	–155.81^i^ ^,^ ^32^
	cc-pV*X*Z^32^	5	–155.62	±**0.36**	±0.81	±1.4	0.19	
		6	–155.73	±**0.08**	±0.17	±0.28	0.08	
HF molecule	CCSD (singlet pairs)	5	–213.72	±**0.61**	±1.4	±2.3	0.58	–213.14^i^ ^,^ ^32^
	cc-pV*X*Z^32^	6	–213.34	±**0.26**	±0.57	±0.95	0.20	
F_2_ molecule	CCSD (singlet pairs)	5	–414.83	±**1.9**	±4.2	±6.9	0.67	–414.16^i^ ^,^ ^32^
	cc-pV*X*Z^32^	6	–414.44	±0.26	±**0.58**	±0.97	0.28	

a A brief description of the data and level of theory is given in the first and second columns, respectively. The maximum cardinal number, *X*_max_, used in the procedure is given in the third column. In the fourth column, the result *e*_*X*_max__ extrapolated using basis sets (*X*_max_, *X*_max_ – 1) is shown. The determined error bars at the 1*σ*, 2*σ*, and 3*σ* confidence levels (see the text for precise definitions) are given in the fifth, sixth, and seventh columns, respectively. The reference result is given in the last column, while the absolute deviation of a given result from the corresponding reference data in the second to last column. The most narrow confidence interval that correctly predicts the difference from the reference result is shown in bold. All results are given in mHa.

b Hartree–Fock, numerical solution on a grid; FCI, iterative free-complement calculations within the generalized Hylleraas basis.

c Explicitly correlated Gaussian calculations with 3600 basis set functions.

d Explicitly correlated MP2 with the Hylleraas basis for expansion of pair functions.

e Explicitly correlated FCCD with the Hylleraas basis for expansion of pair functions and two orbital-based corrections.

f Hartree–Fock, polarization-consistent basis sets and extrapolation; FCI, explicitly correlated Gaussians with 900 functions.

g Explicitly correlated Gaussian basis with 350 functions for expansion of pair functions.

h Finite element MP2 calculations and angular momentum extrapolation.

i Average of CBS-extrapolated orbital calculations with cc-pV*X*Z and *X*ZaP basis sets with *X* = 9 and 10.

j MP2-R12/B and CCSD-R12/B calculations with uncontracted basis sets 19s14p8d6f4g3h for C, O, and F and 9s6p4d3f for H.

**Table 3:** Same as Table 2 but for Properties Other Than Atomic and Molecular Energies^a^

				confidence intervals				
system and quantity	method and basis	*X*_max_	*e*_*X*_max__	1σ (68.27%)	2σ (95.45%)	3σ (99.73%)	error	reference value
HF molecule	CCSD(T)	5	187.54	±**0.94**	±2.1	±3.5	0.23	187.32 ± 0.13^a^ ^,^ ^67^
atomization energy (kJ/mol)	aug-cc-pCV*X*Z^67^	6	187.35	±**0.13**	±0.30	±0.49	0.03	
		7	187.33	±**0.01**	±0.03	±0.04	0.01	
N_2_ molecule	CCSD(T)	5	472.05	±0.54	±1.2	±**2.0**	1.54	470.51 ± 0.10^a^ ^,^ ^67^
atomization energy (kJ/mol)	aug-cc-pCV*X*Z^67^	6	471.26	±0.53	±**1.2**	±2.0	0.75	
		7	470.99	±0.19	±0.41	±**0.68**	0.48	
AlH_3_ molecule	CCSDT(Q)	4	214.699	±**0.813**	±1.829	±3.047	0.774	213.925^b^ ^,^ ^68^
atomization energy (kcal/mol)	cc-pV*X*Z^68^	5	214.209	±**0.325**	±0.730	±1.216	0.284	
CS molecule	CCSDT(Q)	4	170.957	±**2.821**	±6.348	±10.575	0.554	171.512^b^ ^,^ ^68^
atomization energy (kcal/mol)	cc-pV*X*Z^68^	5	172.036	±**0.714**	±1.608	±2.680	0.525	
HCl molecule	CCSDT(Q)	4	107.447	±**0.740**	±1.666	±2.774	0.080	107.367^b^ ^,^ ^68^
atomization energy (kcal/mol)	cc-pV*X*Z^68^	5	107.664	±0.144	±**0.323**	±0.537	0.297	
P_2_ molecule	CCSDT(Q)	4	116.414	±**3.965**	±8.923	±14.880	0.348	116.762^b^ ^,^ ^68^
atomization energy (kcal/mol)	cc-pVXZ^68^	5	117.166	±**0.498**	±1.120	±1.866	0.404	
helium dimer	FCI	5	11.097	±**0.20**	±0.45	±0.75	0.096	11.001^c^ ^,^ ^69^
interaction energy (K)	d*X*Z^69^	6	10.986	±**0.074**	±0.17	±0.28	0.015	
internuclear distance of 5.6 au		7	10.968	±0.012	±0.027	±**0.045**	0.033	
helium dimer	FCI	5	3771.15	±**3.8**	±8.4	±14	3.78	3767.73^c^ ^,^ ^69^
interaction energy (K)	d*X*Z^69^	6	3766.72	±**3.2**	±7.2	±12	1.01	
internuclear distance of 3.0 au		7	3766.04	±0.46	±1.1	±**1.7**	1.69	
benzene dimer	MP2	4	9.265	±**0.43**	±0.97	±1.6	0.028	9.293^d^ ^,^ ^70^
interaction energy (kcal/mol)	A′V*X*Z^70^	5	9.302	±**0.025**	±0.057	±0.094	0.009	
argon dimer	CCSD(T)	5	97.294	±**0.76**	±1.7	±2.8	0.151	97.445 ± 0.063^e^ ^,^ ^71^
interaction energy (cm^–1^)	d↑↓-disp-*X*Z + midbond^71^	6	97.515	±**0.15**	±0.33	±0.55	0.070	
He atom	FCI	4	1.383061	±**0.00022**	±0.00049	±0.00082	0.000131	1.383 192^f^ ^,^ ^72^
dipole polarizability (au)	d*X*Z^52^	5*	1.383096	±0.000023	±0.000052	±0.000086	0.000097	
		6	1.383147	±0.000035	±**0.000077**	±0.00013	0.000045	
		7	1.383170	±0.000015	±**0.000034**	±0.000057	0.000022	
H_2_ molecule	FCI	3	6.38944	±**0.0068**	±0.016	±0.026	0.00212	6.387 32^g^ ^,^ ^73^
dipole polarizability (au)	aug-mcc-pV*X*Z^52^	4	6.38731	±**0.0015**	±0.0032	±0.0053	0.00001	
		5	6.38772	±0.00028	±**0.00062**	±0.0011	0.00041	
Ne atom	ΔCCSD(T)	7	–33.431	±0.13	±**0.28**	±0.46	0.166	–33.265 ± 0.003^h^ ^,^ ^7^
dipole polarizability (10^3^ au)	q-aug-*n*ZP′^7^	8	–33.357	±0.050	±**0.12**	±0.19	0.092	
		9	–33.315	±0.028	±**0.063**	±0.11	0.050	
		10	–33.300	±0.010	±0.023	±**0.038**	0.035	
		11	–33.289	±0.008	±0.017	±**0.027**	0.024	
Ar atom	ΔCCSD	4	–0.3794	±**0.14**	±0.31	±0.51	0.0152	–0.3642 ± 0.0004^i^ ^,^ ^10^
dipole polarizability (au)	da*X*Z^10^	5	–0.3536	±**0.018**	±0.039	±0.064	0.0106	
		6	–0.3620	±**0.0056**	±0.013	±0.021	0.0022	
		7*	–0.3622	±0.0002	±0.0003	±0.0005	0.0020	
		8	–0.3633	±0.0008	±**0.0016**	±0.0027	0.0009	

a The units are given in the first column in each case.

b Orbital calculations with aug-cc-pCV*X*Z basis sets with *X* = 7 and 8 and extrapolation to the complete basis set limit.

c Extrapolated from the cc-pV*X*Z basis set pair with *X* = 5 and 6.

d Explicitly correlated Gaussians calculations with up to 2400 basis set functions.

e Extrapolated from the A′V*X*Z basis set pair with *X* = 5 and 6.

f Recommended best estimate obtained as the average of extrapolations from four different basis set sequences.

g Basis of 900 correlated exponential functions with randomly generated complex exponents.

h Basis of 80 and 65 correlated exponential functions for the ground and response wave functions, respectively.

i Extrapolated from q-aug-*n*ZP′ with *n* = 11 and 12 basis set pair.

j Extrapolated from da*X*Z with *n* = 8 and 9 basis set pair.

For the purpose of further analysis, we call the uncertainty prediction successful at a given confidence level if the true error evaluated against the reference data falls within the determined error bars. Gathering all atoms and/or molecules, properties, and basis set combinations included in Tables 2 and 3, we have 79 distinct sets of data to which the proposed uncertainty prediction procedure was applied. Of those, uncertainty prediction at the 1σ level is found to be successful in about 57% of cases and at the 2σ level in 84% of cases. We have encountered only two cases in which the 3σ level is unsuccessful (denoted by asterisks in Table 3), and we will discuss these examples in detail further below. First, let us put the obtained results into perspective by comparing these percentages with two other popular schemes for attaching uncertainty to the extrapolated results. The first is the difference between two consecutive extrapolated results, i.e.,|*e*_*X*_ – *e*_*X*–1_|, while the second is the difference between the extrapolated result and the corresponding result in the largest basis set available, i.e., |*e*_*X*_ – *E*_*X*_|. The first method is successful only in about 26% of cases considered in Tables 2 and 3, so clearly, it is not a reliable indicator of the residual basis set incompleteness error. The second method is successful in most cases considered in Tables 2 and 3, but the error bars determined in this way are usually very broad. Therefore, the use of this approach leads to gross overestimation of the actual error, making it a much less attractive method in practice.

Returning to the examples in which error prediction at the 3σ level is not successful, the origin of the problem is traced back to the violation of the fundamental assumption of our method, namely the monotonic decrease in the absolute differences between extrapolated results. Taking the polarizability of the argon atom as an example, the extrapolated results in this case are −0.3620, −0.3622, and −0.3633 for *X* = 6, 7, and 8. Clearly, the difference between *e*_6_ and *e*_7_ is smaller here than that between *e*_7_ and *e*_8_, violating the assumptions from eqs 4 and 5. One could argue that in such situations the proposed method should not be used at all or a different extrapolation scheme should be applied to eliminate this pathological behavior. However, we propose a simple modification of the procedure in such situations: use the difference |*e*_*X*_ – *e*_*X*–2_| rather than |*e*_*X*_ – *e*_*X*–1_| to initiate the random walk starting with *e*_*X*_. After this straightforward modification, the result at the 1σ uncertainty level becomes −0.3622 ± 0.0057, and the true error (0.0020) is well within the determined error bars. Using the aforementioned procedure with the second problematic case (helium polarizability), we obtain the value of 1.38310 ± 0.00020 at the 1σ uncertainty level with the true error being equal to 0.00010.

However, the success of the modified procedure in this single case is not sufficient to claim that it performs equally well in general. To address this, we looked for other examples in which the fundamental assumption is violated. A handful of them are found in Tables 2 and 3; however, deviations from monotonicity of *e*_*X*_ are small, and the unmodified procedure predicts the error successfully. However, we encountered significant violations of the fundamental assumption in the interaction energies of the helium dimer taken from refs 55 and 69. For example, for the internuclear distance *R* = 4.17 au, the extrapolated results for *e*_*X*_ are 176.59, 178.60, 178.59, and 178.30 K for *X* = 4, 5, 6, and 7, respectively. Clearly, the middle two numbers are accidentally close to each other, and the differences between the extrapolations do not behave monotonically. As illustrated in Figure 2, this leads to significant underestimation of the uncertainties at *R* = 4.17 au (and, for the same reason, at a handful of neighboring points on the interaction energy curve). When the proposed modification was applied to all points for which nonmonotonic behavior was observed, the problem of underestimated uncertainty was solved (see Figure 2). In the same spirit, the difference |*e*_*X*_ – *E*_*X*_| can be used as an even more conservative initial bound for the next extrapolation in situations in which the value of *e*_*X*–2_ is not available.

**Figure 2:**
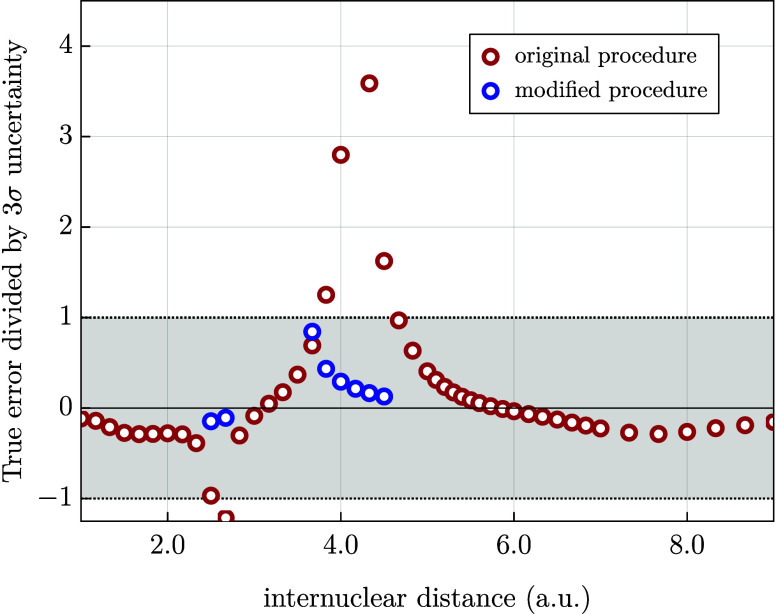
Performance of the original vs modified uncertainty prediction procedure for the interaction energy of the helium dimer (FCI method) as a function of the internuclear distance. The progression of basis sets d*X*Z with *X* = 5, 6, and 7 is used to initiate the random walks. On the vertical axis we show the ratio of the true error of the extrapolation (with respect to the reference 55,69 ) and the uncertainty predicted at the 3σ confidence level. The region in which the uncertainty prediction is considered successful (corresponding to the ratio within the interval [−1, 1]) is shaded gray. The uncertainties were determined using the original (red circles) and modified procedures (blue circles) (see text).

Finally, we observe that the 1σ confidence interval performs particularly well when applied to results obtained from three smallest basis sets (*X* = 2, 3, and 4). Indeed, in Tables 2 and 3, we find 19 separate cases to which the proposed method was applied employing the *X* = 2, 3, and 4 basis set progression. Of them, the error estimate obtained at the 1σ confidence level is successful in 18 (or ≈95%) of cases, a significantly larger success rate than one would expect from the stated probability (68.27%). This may be a consequence of the fact that in smaller basis sets, *X* = 2 in particular, the sources of error other than the lack of higher angular momentum functions remain significant. Insufficient radial saturation, i.e., small number of functions for angular momenta included in the basis, may be the major contributing factor here. While these secondary sources of error typically converge faster as a function of the basis set size, they are effectively extrapolated according to the *X* ^–3^ rule, leading to their slight overestimation.

An important point related to the proposed procedure is how to estimate the total error of theoretical calculations in a situation in which the final result is assembled as a sum of several corrections calculated at different levels of theory. A typical example of such a procedure is the determination of post-CCSD­(T)^74,75^ contributions to some quantity such as atomization energy or bond dissociation energy. In this case, the final theoretical result would be obtained by summing a set of post-CCSD­(T) corrections calculated as a difference between CCSDT^76,77^ and CCSD­(T) calculations, between CCSDT­(Q)^78^ and CCSDT calculations, and so on. The practical reason for splitting the post-CCSD­(T) effects in this way is the observation that higher-order terms usually quickly decrease in magnitude, and it is sufficient to compute them using a smaller basis set. However, as the various post-CCSD­(T) corrections may exhibit different convergence rates,^68^ extrapolating them separately to the complete basis set limit may significantly improve the results. While the procedure proposed in this work can be straightforwardly applied to each post-CCSD­(T) component separately, it is not clear how to determine the error bars of their sum, which is the main quantity of interest. The simplest solution to this problem is to adopt the commonly used assumption that all post-CCSD­(T) components are uncorrelated variables in a statistical sense. The total error is then obtained by calculating the sum of squares of individual uncertainties (obtained at the same uncertainty level) and taking the square root, which follows from the conventional error propagation techniques. We illustrate this in Table 4, where we consider post-CCSD­(T) contributions to the bond dissociation energy of the C_2_ molecule (^1^Σ_g_^+^ ground state) using the results reported by Karton in a recent paper.^79^

**Table 4:** Estimation of the Extrapolation Error of Post-CCSD­(T) Contributions to the Bond Dissociation of C_2_ Using the Procedure Described in This Work^a^

post-CCSD(T) contribution	recommended value	uncertainty
CCSDT-CCSD(T)	–2.268	±0.028
CCSDT(Q)-CCSDT	3.420	±0.008
CCSDTQ-CCSDT(Q)	–1.151	±0.003
CCSDTQ(P)-CCSDTQ	0.412	±0.020
total	0.413	±0.036

a The total uncertainty (in the last row) was calculated as the square root of the sum of squared uncertainties of each post-CCSD­(T) contribution. The extrapolated values were taken from ref 79. All results are given in kcal/mol.

The results presented above were based on the extrapolation scheme of Helgaker et al.^44,45^ However, other extrapolation techniques are also frequently used in the literature, and it is interesting to compare their respective uncertainties predicted by the proposed method. To this end, we selected four distinct two-point extrapolation schemes: (1) *X* ^–3^ method of Helgaker et al.^44,45^ (same as above), (2) (*X* + 1/2)^−4^ scheme of Martin,^80^ (3) method based on the Riemann ζ function,^52^ and (4) scheme proposed by Varandas in which parameter *X* characterizing the basis set size is a non-integer.^33,35,81^ For the purpose of this test, we return to the same systems and basis set combinations as in Table 1 and perform analogous calculations using the four extrapolation methods. In extrapolation scheme 4, the hierarchical numbers for the V*X*Z and AV*X*Z basis sets^81^ were used in test case 1 and test case 2, respectively. The results are included in Table 5 at the 1σ, 2σ, and 3σ confidence levels.

**Table 5:** Comparison of Uncertainties Assigned to the Extrapolated Results Based on Four Different Extrapolation Methods^a^

extrapolation method	test case 1 (H_2_ molecule)	test case 2 (carbon atom)
1σ (68.27%)		
*X* ^–3^	40.8378 ± 0.0078	154.7 ± 2.2
(*X* + 1/2)^−4^	40.824 ± 0.012	154.0 ± 2.2
Riemann ζ	40.8455 ± 0.0026	155.7 ± 1.1
non-integer *X*	40.828 ± 0.013	154.2 ± 1.1
2σ (95.45%)		
*X* ^–3^	40.838 ± 0.018	154.7 ± 4.8
(*X* + 1/2)^−4^	40.824 ± 0.027	154.0 ± 5.0
Riemann ζ	40.8455 ± 0.0058	155.7 ± 2.5
non-integer *X*	40.828 ± 0.028	154.2 ± 2.4
3σ (99.73%)		
*X* ^–3^	40.838 ± 0.029	154.7 ± 7.9
(*X* + 1/2)^−4^	40.824 ± 0.045	154.0 ± 8.3
Riemann ζ	40.8455 ± 0.0096	155.7 ± 4.1
non-integer *X*	40.828 ± 0.047	154.2 ± 3.9
reference	40.8463	156.287

a See the text for more details. All values are given in mHa (with signs reversed for the sake of convenience).

The data reported in Table 5 lead to the conclusion that all extrapolation schemes give consistent results, if their respective uncertainties are taken into account. Even if we consider a pair of extrapolation schemes that differ the most from each other (40.824 vs 40.846 for test case 1; 154.0 vs 155.7 for test case 2), the differences are smaller than the sum of their uncertainties at the 2σ level, 0.033 and 7.5, respectively. Simultaneously, for all data points in Table 5, the differences between the extrapolated results and the corresponding reference values are smaller than the uncertainty at the 2σ level.

In summary, we have introduced a method of estimating the uncertainty of a result obtained through extrapolation to the complete basis set limit. The method is based on an ensemble of random walks that simulate possible extrapolation outcomes that could have been obtained if results from larger basis sets had been available. The ensemble of independent random walks is then analyzed statistically, enabling an uncertainty prediction at a given confidence level. The method is free of empiricism and can be used in conjunction with any extrapolation scheme. Numerical tests performed in this work show that the proposed method is successful in predicting the extrapolation error, leading to error bars that are tight yet conservative at the same time. While the extrapolation error is the natural target for the proposed procedure, it is possible that similar ideas can be used to determine uncertainties due to other sources of error in quantum-chemical calculations and beyond.

## Data Availability

A Python implementation of the proposed procedure is available on GitHub (https://github.com/lesiukmichal/extrapolation-random-walk).^82^
